# Toxicity and autophagy effects of fluorinated cycloplatinated(II) complex bearing dppm ligand on cancer cells in *in-vitro* culture and *in-silico* prediction

**DOI:** 10.22099/mbrc.2023.44705.1781

**Published:** 2023

**Authors:** Zahra Kamalzade, Elham Hoveizi, Masood Fereidoonnezhad

**Affiliations:** 1 Department of Biology, Faculty of Science, Shahid Chamran University of Ahvaz, Ahvaz, Iran; 2 Department of Medicinal Chemistry, School of Pharmacy, Ahvaz Jundishapur University of Medical Sciences, Ahvaz, Iran

**Keywords:** Autophagy, Cycloplatinated(II) Complex, mTOR Pathway, Scaffold, Tumor Cells

## Abstract

Toxicity and autophagy effects of a new complex of platinum II (CPC) were evaluated on HeLa cells cultured on a PCL/gelatin electrospinning scaffold. HeLa cells were treated with CPC on the first, third, and fifth days and the concentration of IC_50_ was determined. The autophagic and apoptotic effects of CPC were examined by MTT assay, Acridine Orange, Giemsa, DAPI, MDC, real-time PCR, Western blot testing, and molecular docking. The cell viability was obtained on days 1, 3, and 5 as much as 50, 7.28, and 19%, respectively with a concentration of IC_50_ (100μM) of CPC. The staining results indicated that the treatment of HeLa cells with CPC had antitumor and autophagic effects. Results of RT-PCR showed that the expression of *BAX*, *BAD*, *P53*, and *LC3* genes was significantly increased in the sample treated with IC_50_ concentration compared to the control sample whereas the expression of *BCL2*, *mTOR*, and *ACT* genes in cells was significantly decreased compared to the control group. Also, these results were confirmed by Western blotting. The data indicated the induction of apoptotic death and autophagy in the studied cells. The new compound of CPC has antitumor effects.

## INTRODUCTION

Cancer is a disease in which the body's cells diverge and multiply abnormally in a malignant tumor and destroy healthy tissue. Cervical cancer is the fourth most common cancer in women, reaching 528,000 new cases and 266,000 annual deaths worldwide. Despite the advancements in screening and treatment strategies, a significant statistics of patients with cervical cancer die [1]. Most cancers today have no definitive cure, but surgical, radiotherapy, chemotherapy, hormone therapy, bone marrow transplantation, etc. are used to prevent their growth [2]. 

A class of medicines used to treat cancer is alkylating agents. DNA alkylation in the nucleus is one of the major reactions leading to cell death and has a long history of clinical applications. Cycloplatinated(II) Complexes are inorganic metal complexes that have a mechanism similar to alkylating agents. This compound binds to DNA through inter-strand cross-links. Several reports have shown that the binding of platinum complexes to the guanine nitrogen 7 disrupts DNA replication and cell death [3, 4]. 

Studies have shown that many anti-cancer compounds and medicines have anti-cancer effects through alterations in gene expression and activation of cell death pathways through apoptosis and autophagy. Apoptosis is a critical component of various processes including normal cell turnover, proper development and function of the immune system, hormone-dependent atrophy, fetal growth, and cell death [5]. Unbalanced apoptosis (too little or too much) is responsible for many human diseases, such as neurodegenerative disease, ischemic injury, autoimmune disorders, and many cancers [6]. 

Nowadays, researchers are also studying cellular mechanisms to find new and effective methods for cancer. One of the critical cellular processes that have attracted the attention of cancer researchers today is autophagy. Activation of autophagy acts as a cellular control mechanism by destroying and removing damaged cellular elements, intracellular pathogens, apoptotic cells, and long-lived proteins [7]. In addition to restoring cellular components, autophagy is involved in the differentiation and remodeling of various organ tissues. Therefore, it is essential to know the potential of cell survival or death for therapeutic potential [8]. As mentioned, 3D culture has provided good conditions today to test new anticancer medicines. Scaffolds are material-based structures in the extracellular matrix that are often produced *in vitro* in a variety of techniques, particularly by electrospinning [9]. 

One of the synthetic polymers made in this way is the polycaprolactone (PCL) scaffold. PCL scaffolds provide a suitable substrate for cell junction and growth. The nano diameter of PCL fibers is an important advantage of this scaffold, which results in the production of scaffolds similar to the body's physiological conditions. Low fiber thickness gives more surface area than fiber volume. Moreover, the high pores and their junction with each other are significant advantages of this type of scaffold. Also, the potential for its integration with other polymers is too important. It is one of the essential features for cell junction and proliferation on the scaffold [10 ,11]. Therefore, it can be stated that the prevalence of cancer in Iran and the world and the importance of cell death processes such as apoptosis and autophagy are based on the use of anticancer agents such as alkylating agents. In this study, autophagic and apoptotic effects of a fluorinated cloplatinated (II) [Pt(dfppy)(dppm)]Cl complex(CPC) were investigated on human cervical cancer cell line (HeLa) cultured on PCL/gelatin electrospinning nanofiber scaffold.

## MATERIALS AND METHODS

**Figure 1:**
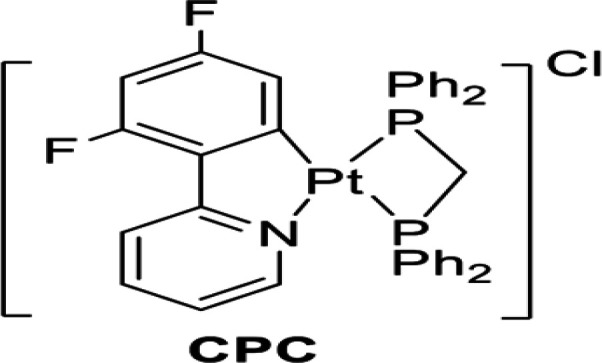
Schematic structures of [Pt (dfppy)(dppm)] Cl complex; (dfppy=2-(2,4-difluorophenyl) pyridine, and dppm = (Bis (diphenylphosphino) methane)

**Chemistry:** The chemical structure of the studied fluorinated cloplatinated(II) [Pt(dfppy)(dppm)] Cl complex (CPC) is shown (Fig. 1). It was synthesized by known published methods [12]. The complexes were previously characterized using different spectroscopies, and here only ^1^H NMR spectroscopy was used to determine its successful formation.

**Cell culture** **: **In this research, the HeLa cancer cells were purchased from the Pasteur Institute of Tehran and proliferated in incubation conditions (Sina Co., Iran) at 37°C, 5% carbon dioxide. HeLa cells were treated with CPC [13] at concentrations of 1000μm, 100μm, 10μm, and 1μm after being cultured on PCL/gelatin scaffolds (3 iterations were considered for each concentration). Also, 3 wells of control samples were considered for each concentration in the plate. The treated cell plate was incubated for 24h to be used for cell morphology and IC_50_ assay. Cells were then treated with IC_50_ concentrations and cell viability was surveyed on days 1, 3, and 5.

**Preparation of scaffolds for scanning electron microscopy** **:** In this study, HeLa cells were cultured on a PCL/gelatin scaffold on a 24-well plate for SEM photography. The incubation and fixation of the scaffold for SEM photography were conducted after 24 hours. The medium on each well was gently removed and washed, and 2.5μL of 2.5% glutaraldehyde solution was added to each well. They were kept at room temperature for 2 hours and the dehydrations were performed with increasing concentrations of alcohol (30, 50, 60, 70, 80, 90, 100, 100%). The plate was then stored at -20°C for 2 hours and freeze-drying was done to dry and dehydrate the scaffold. Finally, SEM photography (LEO 1455VP, Japan) was performed using a gold coating.

**Observation and morphology of HeLa cells: **HeLa cells were cultured in 96-well plates, 1 × 10^4^ per well. Moreover, the 24-hour treatment of these cells was performed with the IC_50_ concentration of CPC. Cell morphology changes were then observed under an inverted microscope (Biored, USA).

**Staining with Giemsa** **: **For this type of staining, a 4% staining solution was prepared, passed through Whitman No. 1 filter paper. Then Giemsa fixator with a 1:3 ratio of acetic acid to methanol was prepared. Then it follows the Giemsa's staining protocol. The treated and control cells were fixed with a fixator after 24h for 3–5 min. The stain solution was added for 15 to 20 minutes after washing. It was then washed with PBS for 1 minute and placed under an inverted microscope for photography.

***Ethidium Bromide/Acridine Orange stain*:** Acridine orange/ethidium bromide staining is one of the common staining methods to check cell survival. So, in this double staining, live cells are seen in green and dead cells are seen in orange. In this method, cells were first incubated on the PCL/gelatin scaffold for 24h. Cells were then incubated with IC_50_ of CPC for 24h. The supernatant was then removed from the well and the acridine orange/ethidium bromide solution was added at a concentration of 100 μg ml^-1^. After 5 minutes, they were observed and photographed with a fluorescent microscope (Olympus, Japan).

**MTT assay:** Cell plates were incubated to evaluate cell viability by MTT assay. The supernatant was removed with the medicine and washed with PBS. Then 100 µm of MTT solution with a concentration of 0.5 μg ml^-1^ was added to each well. The plate was incubated for 3 to 4 hours. After this period, the extracts were extracted and 100µl of DMSO (dimethyl sulfoxide) was added to each well. After that, each well was slowly pipetted and incubated for 15 minutes. Afterward, the plate was mounted in an ELISA reader (Fax 2100, USA) and the optical absorption was read at 570 nm. Then bioactivity and IC50 concentration was analyzed.

**DAPI** **stain** **ing: **The DAPI staining solution (2-phenyindele-4,6-diamidino) was used, which is a fluorescent dye. The cells were first washed with PBS and fixed in 4% formaldehyde and incubated for 20 min. The cells were washed again with PBS and exposed to DAPI at a concentration of 1μg ml^-1^ for 5 minutes. Finally, they were examined by fluorescent microscopy, cell nucleus morphology, and changes in the treatment.

**MDC** **stain** **ing: **The dye solution was prepared at a concentration of 50 μM in PBS. They were then incubated for 45 minutes to 1 hour on control cells and cells treated for 24 hours. After this, cells were washed 3 times with PBS and observed by a fluorescent microscopy.

**Real-time PCR assay: **Real-time PCR was used to investigate the effects of CPC on apoptosis and autophagy induction and expression of genes involved in apoptosis and autophagy. Initially, the sample was prepared so that two wells were provided for control cells in a 6-well plate and 2 wells were treated for the treated cells. 250,000 were placed and 2 ml of media added to each well. After 24 hours, the cells in the treatment group were exposed to CPC IC_50_ for 24 hours. 

RNA extraction was then performed using the sineagin kit according to its protocol. Trizol-lysed cell extract was centrifuged twice in chloroform for 15 min at 12,000 rpm in 4℃ and washed with isopropanol. Finally, the solution was dissolved in 75% ethanol and centrifuged at 7500 rpm for 8 minutes, then dried and the resulting RNA was dissolved in water. DNA contaminations were eliminated using RNase-free DNase. Then, it was transferred to a nanodrop machine to measure the concentration and purity of the sample. The samples with an absorbance ratio of 280/260 nm were selected for cDNA synthesis in the range 1.7 to 2. The Hypascript first stranh synthesis kit was then used for the cDNA synthesis. The kit included all of the items as a Master Mix, which was eventually delivered to a final volume of 20µl (microliter). According to the kit protocol, 1μg of purified RNA with the Random Hexamer Primer mixed with dNTP mixture and for 5 min at 65℃, then incubated and quickly transferred onto the ice. Thereafter, buffer, ribonuclease inhibiting protein, and the enzyme was added and placed in a thermocycler. 

In this study, the final real-time PCR assay was performed using the Solis BioDyne kit, in which cyberlein dye was used as a fluorescent material and the GAPDH gene was used as the internal control. The final step of RT-PCR was performed using RUSH after centrifugation of cDNA to amplify the desired DNA fragment. In addition, specific gene primers were designed using the NSBI site (Table 1).

**Table 1:** Primer sequence for qRT‐PCR

**Name**	**Primer Sequence (5'→3')**
GAPDH (F)	GCAAGAGCACAAGAGGAAGA
GAPDH (R)	ACTGTGAGGAGGGGAGATTC
BAX (F)	GCTGGACATTGGACTTCCTC
BAX (R)	ACCACTGTGACCTGCTCCA
BAD (F)	CGGAGGATGAGTGACGAGTT
BAD (R)	CCACCAGGACTGGAAGACTC
BCL-2 (F)	GATGGGATCGTTGCCTTATGC
BCL-2 (R)	CCTTGGCATGAGATGCAGGA
mTOR (F)	CGTGCTGGACATCATCCGAG
mTOR (R)	GTGAGGGCAGGGCTTAGTC
AKT (F)	CCATGGACAGGGAGAGCAAA
AKT (R)	CAGCCAACCCTCCTTCACAA
P53 (F)	GGAGGGGCGATAAATACC
P53 (R)	AACTGTAACTCCTCAGGCAGGC
Beclin-1 (F)	ATGGAGGGGTCTAAGGCG
Beclin-1 (R)	TGGGCTGTGGTAAGTAATG
Atg5 (F)	GGACCTTCTACACTGTCCATCC
Atg5 (R)	TGTCATTCTGCAGTCCCATC
LC3 (F)	GATAATCAGACGGCGCTTGC
LC3 (R)	ACTTCGGAGATGGGAGTGGA

**Western blotting:** RIPA was used for the isolation of cell lysis protein using buffer and protein concentration was measured with a BCA protein assay kit (Beyotime). Then, total protein was separated by SDS-polyacrylamide gel electrophoresis. They were then transferred to polyvinylidene fluoride (PVDF) membranes. Samples were blocked with 5% BSA (bovine serum albumin) in PBS containing Tween-20 and 0.05% Tris-buffered saline. Membrane was incubated for 1.5h at room temperature with anti-pAKT (1:500, Abcam), anti-AKT (1:500, Abcam), anti-pmTOR (1:500, Abcam), anti-mTOR (1:500, Abcam), p-AMPK (1:500, Abcam) and GADPH (1500, Abcam) anti-rabbit secondary antibody IgG-HRP (1:11000 Abcam). Finally, the tapes are labeled with DAB (Sigma, German).

**Molecular docking:** To prognosticate the interplay of proteins affected in autophagy, Molegro virtual docker (CLC Bio company, Aarhus, Denmark) was used along with g Molegro Virtual viewer V2.5 software. Three dimensional and crystal structures of the proteins were obtained from the *PDB *database and the structure of CPC as a ligand was drawn using a Chemdraw ultra software. The energy of EFL1 was reported from the last output. To prepare the inputs, we rejected the metal atoms, H2O particles, solvent units, and set the side chain. Docking was done in Surflex-Dock Geom style. The total records were regarded as a steady interplay when the number was more than 5.

**Statistical analysis:** SPSS software was used for statistical analysis. The used tests were one-way ANOVA with post-hoc Tukey. The software Excel 2019 was used to create the graph. A significant difference between the samples was defined as P≤0.05.

## RESULTS

**Figure 2:**
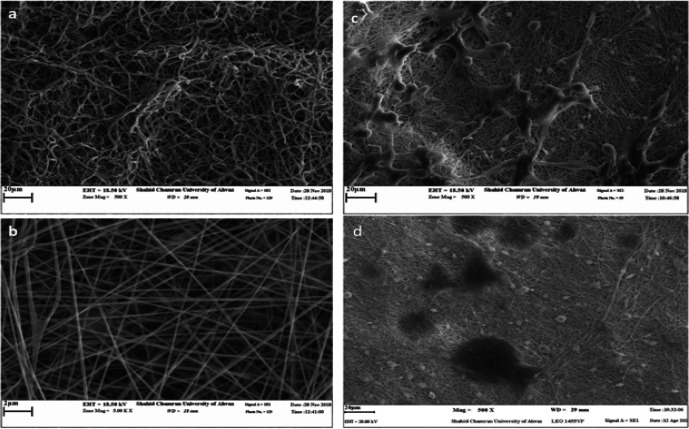
Morphological examination with a microscope, A) Image of PCL/gelatin electrospinning nanofiber scaffold cell-free with scanning electron microscope, B) High-magnification cell-free scaffold. The scaffolds are irregular and heterogeneous and their fibers are uniformly dispersed, C) Image from the morphological investigation of HeLa cells cultured on PCL/gelatin electrospinning nanofiber scaffold by scanning electron microscope, D) Scaffold with the cell with more magnification

Three-dimensional morphology of PCL/gelatin electrospinning nanofiber scaffolds was investigated without cell presence under SEM microscopy. According to the SEM images, the scaffolds are irregular and heterogeneous, the fibers are uniformly dispersed, and did not observe any beads in the scaffolds (Fig. 2A, 2B). The results of morphological observations of HeLa cells cultured on scaffolds are also shown in Fig. 1. The results show that the scaffolds have good mechanical properties and porosity, as well as growth, proliferation, and normal 3D morphology similar to the in vivo conditions of HeLa cells and their association and integrity. The cells are significantly attached to the scaffold and spread evenly over it (Fig. 2C, 2D).

**Figure 3:**
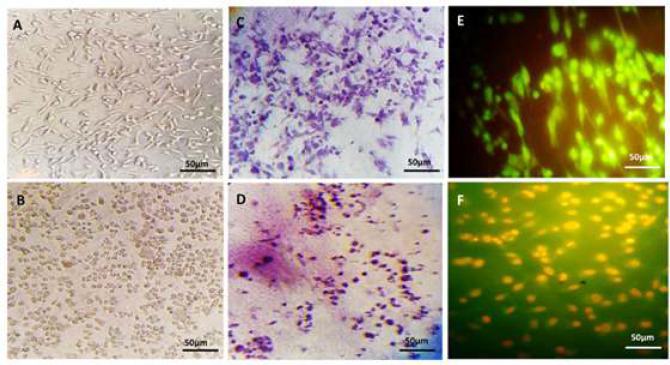
Results of morphological analysis using invert microscopy for HeLa cells, A) Morphology of HeLa cells using an inverted microscope in control sample after 24h, B) Morphology of HeLa cell treated with IC_50_ concentration of CPC for 24h, the shrinkage and reduction of cells, nucleation of the nucleus, reduction of cytoplasm volume, loss of normal cell morphology, and cellular connection and interaction, as well as the appearance of individual cells were seen in the treated sample images, C) Giemsa staining of HeLa cells on PCL/gelatin scaffold, HeLa cell morphology with the control sample in an inverted microscope. D) Giemsa staining of HeLa cells on PCL/gelatin scaffold, HeLa cell morphology with an inverted microscope treated with IC_50_ concentration of CPC. Cell shrinkage, nucleation shrinkage, and chromatin are well visible in these images, E) Evaluation of apoptosis using Acridine orange/ethidium bromide staining of HeLa cells on PCL/gelatin scaffold by a fluorescent inverted microscope of the control sample. The nuclei of the cells are round, coarse, translucent, and green, indicating their viability, F) Evaluation of apoptosis using Acridine orange/ethidium bromide staining of HeLa cells on PCL/gelatin scaffold by a fluorescent inverted microscope of the cells treated with IC50 concentration of CPC

For investigation the morphology, cells were observed under an inverted microscope. Morphological analysis of HeLa cells in both untreated and Giemsa-stained cells using an inverted microscope showed that HeLa cells under 24-hour treatment with IC_50_ concentration of CPC were significantly different from control cells. In the treated cells, shrinkage and decrease in cell size, nucleation of the nucleus, reduction of cytoplasm volume, loss of normal cell morphology, cellular interactions, and interactions were observed. This indicates that these concentrations have a toxic effect and drive the cell to cell death (Fig. 3A-D).

Acridine orange is a color that is absorbed by living cells. It enters the living cell's DNA and gives a green look to the living cell chromatin under the microscope. The cells that have died of cell death are seen orange. In this study, HeLa cells were stained with acridine orange/ethidium bromide for 24h after treatment and incubated to determine their apoptotic morphology. In HeLa cells treated with IC_50_ CPC, the nuclei were individually compared to the control group, where the nuclei were clustered together. The cells of the control group are also round, coarse, translucent, and green, indicating their viability. Cells of the group treated with this compound were treated with a single, compact, and dense orange nucleus confirming the apoptotic cell death in the cell group (Fig. 3 E, 3F).

**Figure 4:**
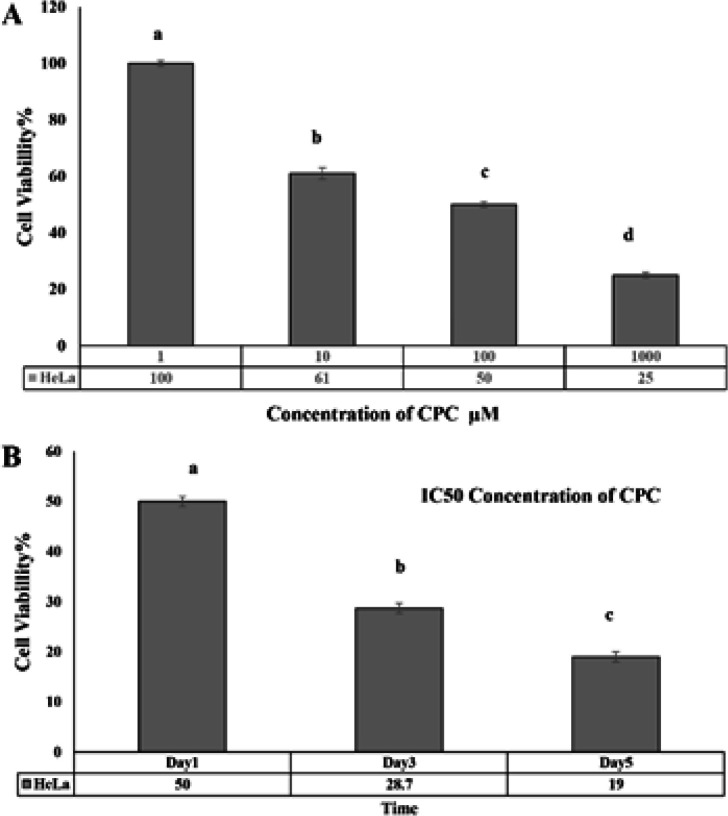
A) Evaluation of cell viability by MTT assay on HeLa cells at concentrations of 1, 10, 100, 1000μM treated with CPC. Here concentration of 100μM of CPC was measured as IC_50_ concentration in HeLa cells in the first day. B) The effect of IC_50_ (100μM) CPC on days 1, 3, and 5 on HeLa cell viability with 3 iterations. The dissimilar letters indicate a significant difference at the confidence level of p˂0.05

MTT assay was performed to investigate the effect of CPC on the HeLa cells at concentrations of 1, 10, 100, 1000μM on the first day. Statistical results showed that biodegradation of HeLa cells after exposure to these doses of the CPC was significantly decreased compared to control. Statistical results showed their viability with the doses expressed as 100, 61, 50, and 25%, respectively. The IC_50_ concentration of this complex on HeLa cells was also determined at a dose of 100μM (Fig. 4). HeLa cells on days 1, 3, and 5 was treated with IC50 (100μM) and compared with the control group. Cell survival rates at days 1, 3, and 5 were 50, 28.7, and 19%, respectively. The percentage of cell survival was lower on day 5 than on day 3 and day 1, and cell survival was lower on day 3 than day 1. This indicates a time-dependent effect of CPC on HeLa cells (Fig. 4).

**Figure 5:**
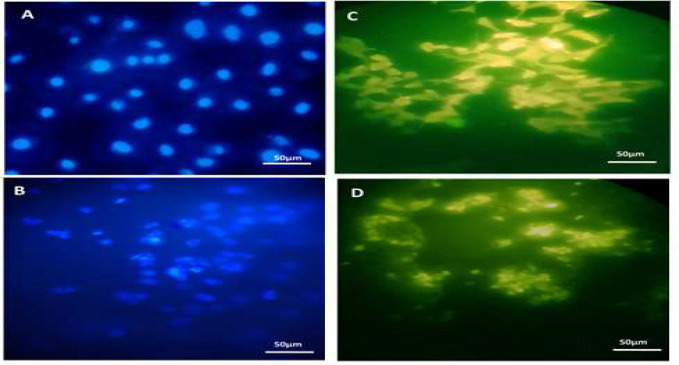
Evaluation of nuclear apoptosis using DAPI staining of HeLa cells and evaluation of autophagy using MDC staining, A) HeLa cell nucleus staining with DAPI on PCL/gelatin scaffold, control sample, B) HeLa cell nucleus staining with DAPI on PCL/gelatin scaffold, the sample treated with IC_50_ concentration of CPC. In the treatment group, the decrease in the volume, shrinkage, and compression of the cell nucleus compared to the large and clear cells of the negative control group confirmed the cell apoptosis, C) HeLa cell nucleus staining with MDC on PCL/gelatin scaffold, control sample. Their cytoplasm is elongated, D) HeLa cell nucleus staining with MDC on PCL/gelatin scaffold, the sample treated with IC_50_ concentration of CPC

After 24h treatment of HeLa cells performed by CPC, the cells were stained by DAPI and the DNA was absorbed into the cell nucleus. Thus, the morphology of the nucleus and their changes were examined by a fluorescent microscope. The nuclei of control cells were transparent, large, and spherical with no density and compression while the presence of wrinkled and dense nuclei indicates apoptotic cell death in the treated cell group (Fig. 5A, 5B). In MDC staining, the cytoplasm becomes stained and the nucleus would not be stained. HeLa cells treated with CPC and incubated for 24h were stained according to the protocol of this procedure and the results are as follows. The cells in the control sample are normal and healthy. Their cytoplasm is elongated, but HeLa cells are formed in samples treated with CPC with IC_50_ concentration and autophagic vacuoles confirm the occurrence of autophagy (Fig. 5C, 5D).

**Figure 6:**
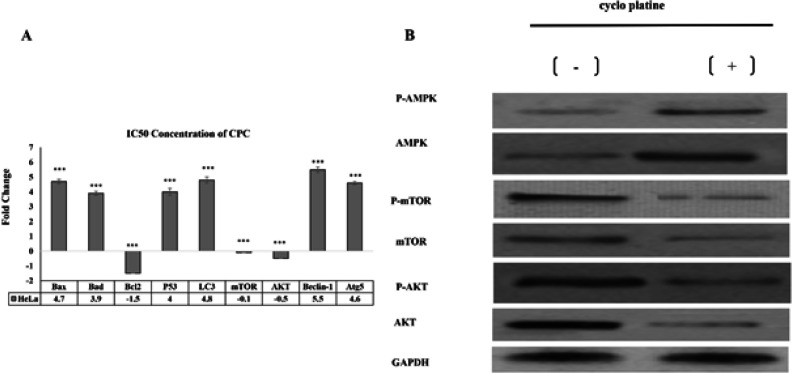
A. Gene expression changes in HeLa cells treated for 24h with IC_50_ concentration of CPC. Increased expression of *Bax*, *Bad*, *p53*, *Beclin1*, *Lc3*, *Atg5* and decreased expression of *Bcl2*, *mTOR*, *AKT* in HeLa cells under 24-hour treatment with CPC compared to the control group indicates the death of autophagic death in cancer cells by this material. *** indicates a significant difference with the control group at p<0.001 level. The fold change control sample was considered as much as +1. Each experiment was iterated three times and the *GAPDH* gene was used as the internal control, B. Expression of p-mTOR and p-AKT in the IC_50_-treated groups of CPC in the HeLa cancer cells was significantly down-regulated compared to the control group

The results of RT-PCR showed that gene expression of *BAX*, *BAD*, *P53*, *LC3*, *Beclin1*, and *ATG5* significantly increased compared to control sample in the sample treated with IC_50_ concentration of CPC while the expression of *BCL2*, *mTOR*, and *AKT* genes in HeLa 24-treated cells showed a significant decrease compared to the control group, which results in induction of apoptotic death as well as autophagy in the studied cells (Fig. 6A).

The Western blotting analysis was performed to determine the effects of CPC on the induction of autophagy in HeLa cells and to identify the proteins involved in this process. As shown in fig. 6.B, the expression of p-mTOR and p-AKT was significantly down-regulated in the IC_50_-treated groups of CPC in the HeLa cells compared to the control group. Moreover, the expression of p-AMPK in the treated samples showed a significant increase compared to the control. These results suggest that the mTOR/AKT/PI3K pathway and AMPK/mTOR pathway are involved in the induction of autophagy by CPC in cancer cells. mTOR protein is a negative regulator of autophagy. AMPK and AKT proteins act in the upstream of mTOR.

**Figure 7:**
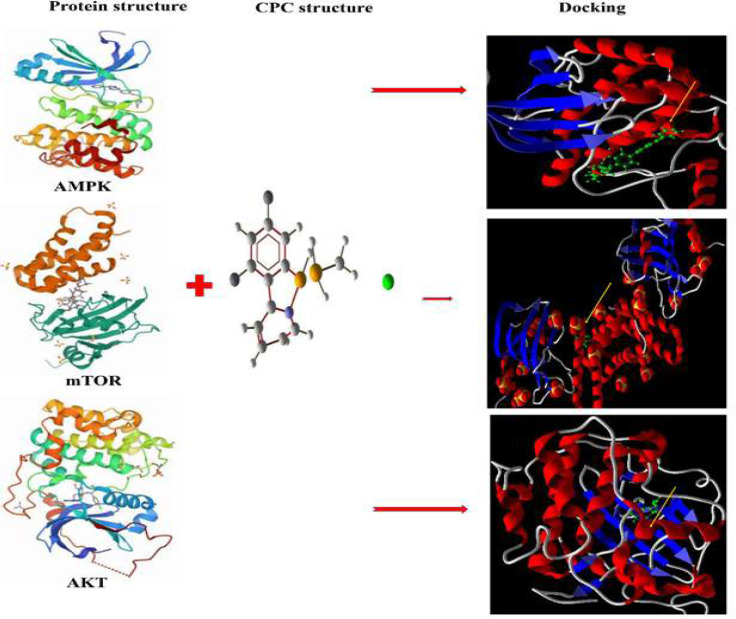
Molecular docking studies and interaction between CPC (green) and mTOR, AMPK, and AKT proteins. Red and blue dashed lines represent steric and hydrogen bonds, respectively. The yellow arrow indicates the docking location

**Figure 8:**
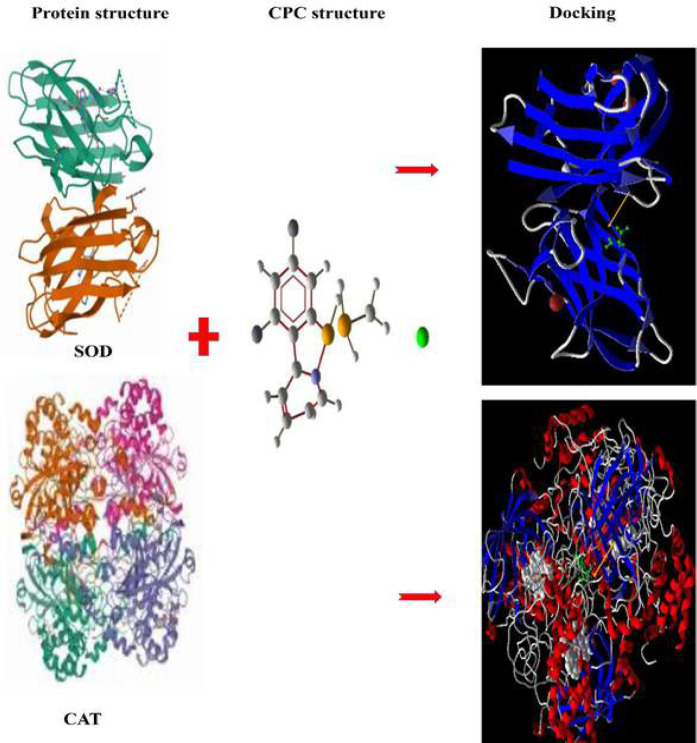
Molecular docking studies, and interaction between CPC (green) and catalase and SOD proteins. Red and blue dashed lines represent steric and hydrogen bonds, respectively. The yellow arrow indicates the docking location

We determined the molecular docking between CPC and some significant organizational proteins, including AMPK, mTOR, AKT, SOD, and catalase through molecular studies. As displayed in Figures 7 and 8 and Table 2, the proteins had considered RMSD<2 and a total score of>5 describing potential interactions within CPC and these samples. CPC caused oxidative stress and autophagy through basic interaction with redox and autophagic proteins. Our data showed that CPC can interact with redox and autophagic proteins. Furthermore, the outcomes confirmed the steric and hydrogen bonds between CPC and proteins.

Theoretically, CPC was attracted with AMKP by formation of hydrogen and steric bonds at Ala180, Ser199, Trp198, Phe102, and Leu212. CPC was attracted with AKT by formation of hydrogen and steric bonds at Phe438(A) and Glu 228(A). CPC was bound to mTOR by the formation of hydrogen and steric bonds at Trp2101(B) (Fig. 7 and Table 2). CPC was bound to catalase through formation of hydrogen and steric bonds at His364(A), His364(B), and Pro70(D). Moreover, CPC was bound to SOD through formation of hydrogen and steric bonds at Asn53(B) and Lys9(A) (Fig. 8 and Table 2).

**Table 2:** Molecular interactions between CPC and human proteins

**Hydrogen and Steric bonds**	**Total Score**	**RMSD(A˚)**	**PDB(ID)**	**Protein**
Phe438(A) and Glu 228(A)	-69.699	1.643	4GV1	AKT
Trp2101(B)	-78.876	1.652	4DRH	mTOR
Ala180, Ser199, Trp198, Phe102, and Leu212	-151.591	1.324	3AQV	AMPK
Asn53(B) and Lys9(A)	-48.215	1.876	3H2	SOD
His364(A), His364(B), and Pro70(D)	-195.122	1.345	1DGB	Catalase

## DISCUSSION

Cancer is a disease caused by an abnormal proliferation of cells. Cervical cancer is a preventable disease, which is the second most common malignancy in women worldwide. After breast cancer in Iran, this malignancy is the most common type of cancer in Iranian women, which is one of the most common causes of female mortality [14]. 

Many anticancer medicines that can reduce pain, extend patient life, prevent metastasis, and temporarily eliminate tumors. Platinum medicines are the most popular among the metal-based medicines. Cisplatin is one of the most widely used medicines in the treatment of cancer, but it has disadvantages that limit its use. New strategies for designing new complexes were developed to address the disadvantages of cisplatin, which have been used as platinum-based anticancer medicines [13, 15]. 

A fluorinated cloplatinated(II) [Pt(dfppy)(dppm)]Cl complex was used in this study and the results of its effects were investigated on the induction of apoptosis and autophagy on cervical cancer cells (HeLa). The results of this research showed that the new compound of the platinum complex had a dose- and concentration-dependent anti-proliferative and cytotoxic effects on HeLa cancer cell survival. In addition, Giemsa, DAPI, Acridine orange/ethidium bromide staining showed apoptotic death in HeLa cells. Previous types of research indicate that platinum complex medicines impose dose- and concentration-dependent anti-proliferative and antitumor effects on a variety of cancers through complex mechanisms. 

Fereidoonnezhad et al. (2017) showed that CPC medicine containing dppf (1,1'-Bis (diphenylphosphino) ferrocene) significantly increases the percentage of apoptotic cells from 60.35% with 5 ml to 89.20 with 30% and reduces the number of viable cells. These results indicate that induction of apoptosis in a dose and concentration-dependent mode [16]. Lyu et al. (2016) synthesized the platinum (II) (1R, 2R) -N1-2-amyl-1,2-diamino cyclohexane complex. The results of the MTT assay showed that these compounds had relatively good toxicity to A549 and HCT-116 cells (IC_50_: 3.32 μM) compared to cisplatin and oxyplatin [17]. Ghani et al. (2012) from the Egyptian University of Cariyev, synthesized a platinum (II) compound mixed with 1H-benzimidazol-2-ylmethyl-N- (4-bromo-phenyl) –amine and investigated its antitumor activity against breast cancer cell line (MCF7). The results indicated that the compound had an IC_50_ of 10.2 μM compared to cisplatin with an IC_50_ of 9.9 μM [18].

The results of this study showed that expression of *BAX*, *BAD*, and *P53* genes as well as decreased expression of *BCL2* gene in HeLa cells treated with CPC were a confirmation to apoptotic death in this cell line. Increased expression of the *LC3* gene (one of the key genes in autophagosome), *ATG5*, *Beclin1. *Also, decreased expression of *AKT* and *mTOR* genes (two genes that are inhibited during autophagy) were indicative of induction of autophagy cell death with apoptosis by platinum on HeLa cells. 

In line with the observation of the present research, Wattanathamsan et al. (2018) stated in a study performed on the mechanism of cisplatin resistance in non-small cell lung cancer cell line NCI-H460 that this medicine induces apoptosis in these cells by inhibiting the *BIRC5* gene, an anti-apoptotic gene. High concentrations of cisplatin also increased Beclin-1, which can lead to increased autophagy activity. Consequently, cisplatin inhibited the growth of NCI-H460 causing genotoxic damage. High concentrations of this medicine alter the expression of apoptotic and autophagy genes [19]. Yang et al. (2019) in a study on the resistance of ovarian cancer cells to a platinum drug targeting the *BCL2* gene stated that Bcl2 is one of the genes regulating apoptosis in response to DNA damage caused by platinum therapy, which does this by regulating genes such as *BAX* and *BAK* [20]. Therefore, regulation of *BCL2* gene expression can induce apoptosis and decrease platinum medicine resistance in ovarian cancer cell line given the fact that apoptosis and autophagy are both highly conserved processes that preserve organizational and cellular identity, respectively. 

Apoptosis performs by removing the damaged or unwanted cells, but maintains cell homeostasis by recycling proteins or intracellular organelles. They are therefore an important protective mechanism for cancer cells in response to chemotherapy. Therefore, the effect of the drug is crucial in the regulation of tumor cell apoptosis and autophagy. However, these two mechanisms do not always have inhibitory roles on each other because they have signaling pathways in their signaling pathways and can cause cell death at the same time. In this cell line from a series of morphological studies with Giemsa staining, DAPI, and Acridine Orange/Ethidium Bromide, it can be understand that treatment with the CPC activates the apoptosis process in the HeLa cells [21]. 

Frezza et al. (2011) synthesized cyclo metalled Organelo Platinum (II) Complexes containing bisphosphine ligands and examined their antitumor activity in the *in vitro* and *in vivo* conditions. Their pharmacological effects for proteosome inhibition and apoptosis induction activity have shown satisfactory results [22]. Moreover, Florea et al. (2011) stated that platinum complexes are used clinically to treat cancers to induce tumor cell death by interfering with transcriptional mechanisms or DNA folding. In addition, cisplatin damages tumors by apoptosis that is mediated by the activation of various signal transduction pathways including calcium signaling, death receptor signaling, and activation of mitochondrial pathways. 

Increased expression of *BAD*, *BAX*, and *P53* gene and decreased expression of *Bcl2* gene in cancer cells treated with platinum complex showed that inhibition of growth and cytotoxic effects of this treatment in cancer cells was done through apoptosis and cell cycle arrest. Significant increases in the expression of *LC3*, *Beclin1*, and *ATG5*, as well as decreased expression of *mTOR* and *AKT* genes in HeLa cells treated with CPC showed the effects of autophagy induction of this medicine along with apoptotic cell death [23]. To this end, Guo et al. (2013) investigated the effect of a mono-platinum on apoptosis-independent autophagy cell death in human ovarian cancer cells. They concluded that Mono-Pt exerts an anticancer effect through autophagic cell death in apoptotic resistant Caov-3 ovarian cancer. This effect was done by increasing the ratio of Lc3-II to Lc3-I and mediated by the AKT1-mTOR-RPS6KB1 pathway and MAPK1 (ERK2)/MAPK3 (ERK1) signaling [24]. 

In addition, a three-dimensional culture, PCL/gelatin electrospinning nanofiber scaffold, was used. Unlike two-dimensional environments such as petri dish, a three-dimensional culture allows cells to grow *in vivo* in all directions, just as conditions exist in the *in vitro* environment. PCL scaffold provide a suitable substrate for cell growth and junction. The nano diameter of PCL fibers is an important advantage of this scaffold, which results in the production of scaffolds similar to the physiological conditions. Consistent with the objectives of this study in applying scaffold, Imamura et al. (2014) compared two-dimensional and three-dimensional culture media in an anti-cancer medicine test on breast cancer cell line. They stated that cells cultured with 2D culture tended to exhibit an excess of the effect of chemotherapy drugs compared to cells cultured on 3D culture. Their results also showed that a 3D culture medium potentially avoids the antitumor effect observed in a 2D culture medium [25].

In this study, molecular docking investigations were achieved on the PCP complex to discover their acting sites and the finest approach for the bases of its binding energies to DNA [12]. The top-ranked binding energies in the result file were regarded as an answer in each run. The reaction with the lower binding energy was regarded as the most suitable docking outcome [13]. A docking verification stage was accomplished by the reaction of the CPC into the three-dimensional configuration of DNA. Typically, RMSD under 2 a was regarded as a successful prediction [16]. The negativity worths of the binding free energies indicate that CPC binds politely to DNA.

Based on the outcomes of Table 2, it can be supposed that the CPC–DNA docking energies are in reasonable congruence with the in vitro toxic effects. The docked sample indicates that the finest proteins based on in vitro results and molecular docking are AKT and SOD. The considerable actively profitable reaction of the docked pose of CPC shows that the dppm group of CPC suits into the minor track of the DNA.

### Acknowledgements:

The authors are thankful to Shahid Chamran University of Ahvaz for providing facilities. grant number: 1400.

### Conflict of Interest:

The authors declare no conflict of interest.
